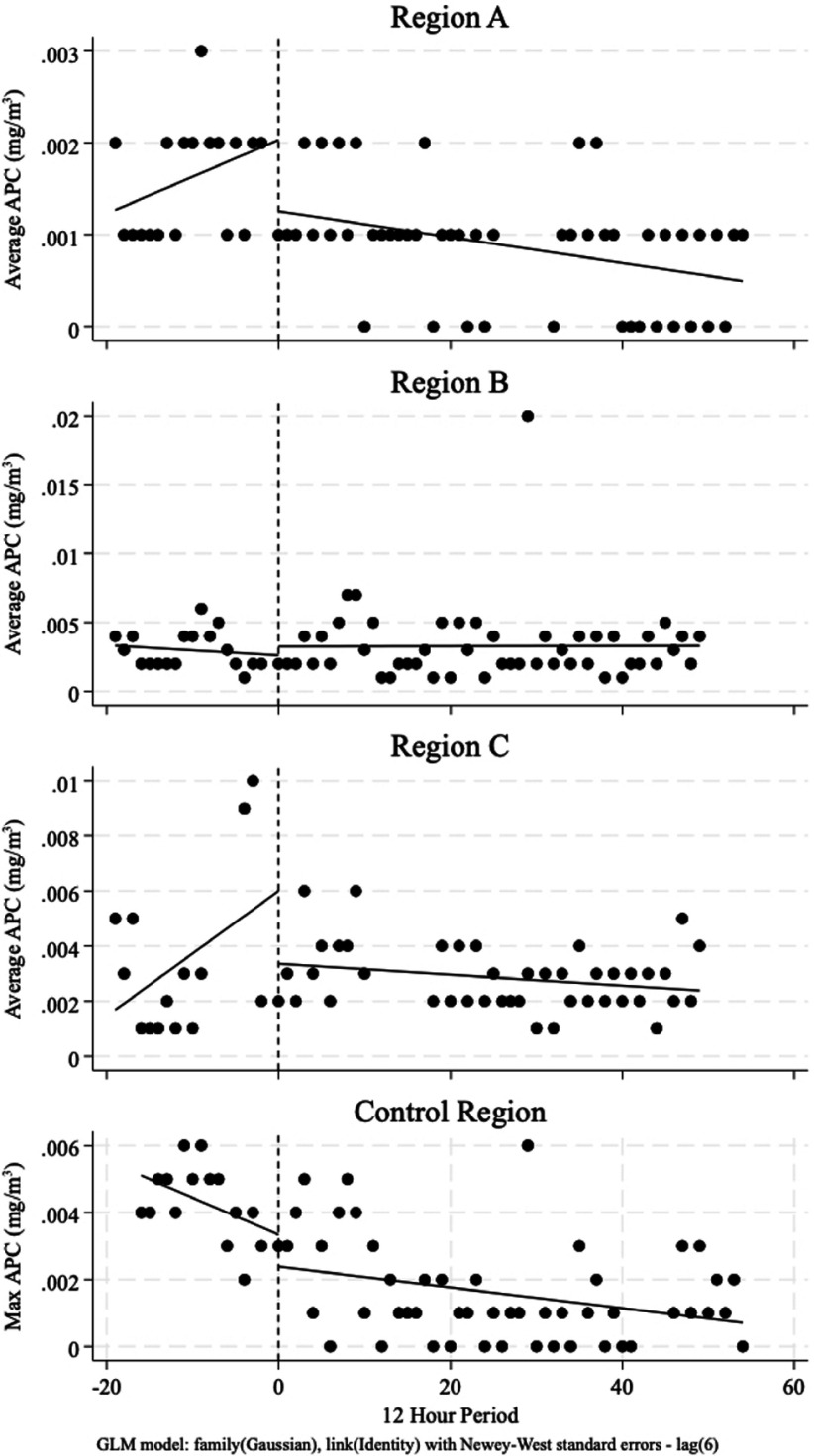# Will Walls Work: An Interrupted Time Series to Model Changes in Dust Burden After Architectural Changes

**DOI:** 10.1017/ash.2025.404

**Published:** 2025-09-24

**Authors:** Nathan L’Etoile, Yun Li, Cody Gladstone, Cindy Hoegg, Amanda Scott, Ericka Hayes, Susan Coffin, Logan Grimes

**Affiliations:** 1 Children’s Hospital of Philadelphia; 2 Children’s Hospital of Philadelphia; 3 Children’s Hospital of Philadelphia; 4 Univ Pennsylvania/Children’s Hospital of Philadelphia; 5 Childrens Hospital of Philadelphia; 6 Children’s Hospital of Philadelphia

## Abstract

**Introduction:** Dust burden in healthcare institutions has been associated with invasive fungal disease (IFD) causing significant morbidity and mortality in immunocompromised patients. Systematically evaluating the impacts of architectural changes on air particulate concentration (APC) could identify risk reduction strategies.

Objectives: We estimated changes in APC in units adjacent to a temporary hard physical barrier in a previously open hospital space after its erection. We propose a model for evaluating the impact of temporary architectural alterations on APC in healthcare settings. **Methods:** Barriers were erected in an open area of a hospital for four weeks. The barrier partitioned the oncology floor from the atrium, which houses the emergency department waiting area. Continuous APCs were measured in multiple locations before and after the barrier was erected. We conducted an interrupted time series on the daily mean and maximum APCs, excluding the period the barrier was being installed. As a control, the same analysis was conducted on a remote location of the hospital. **Results:** A topographical representation of the impacted area is included in Figure 1. Regions A and B are in hallways, adjacent to the barriers, and region C is in a patient care area, separated from the barriers by an automatic door. The control region was the cafeteria, which is separated from the barrier space by several hundred feet. After barrier creation, there was an immediate APC reduction in region A from the predicted mean APC of 2µg/m3 to 1µg/m3 (difference of 0.8 µg/m3, p=0.01) (Figure 2). While the barrier was in place, there was a significant reduction in APC in region A by 0.01µg/m3 per day (p < 0 .001). There was no significant change in APC in regions B and C after the barrier was erected and while it was in place. In the control region, there was no significant change in APC at barrier placement nor afterwards. There was no change in the maximum APC at any of the measurement locations. **Discussion:** Our analysis demonstrated a change in APC at an adjacent area following erection of the barrier; however, APCs were not significantly changed in patient areas. This model could help objectively evaluate changes in particulate concentration. While this analysis cannot predict changes in IFD incidence, it could inform whether permanent architectural changes might reduce APC. **Conclusions:** We propose a model to evaluate changes in APCs from temporary architectural changes, which could inform permanent architectural changes.

**Figure:**
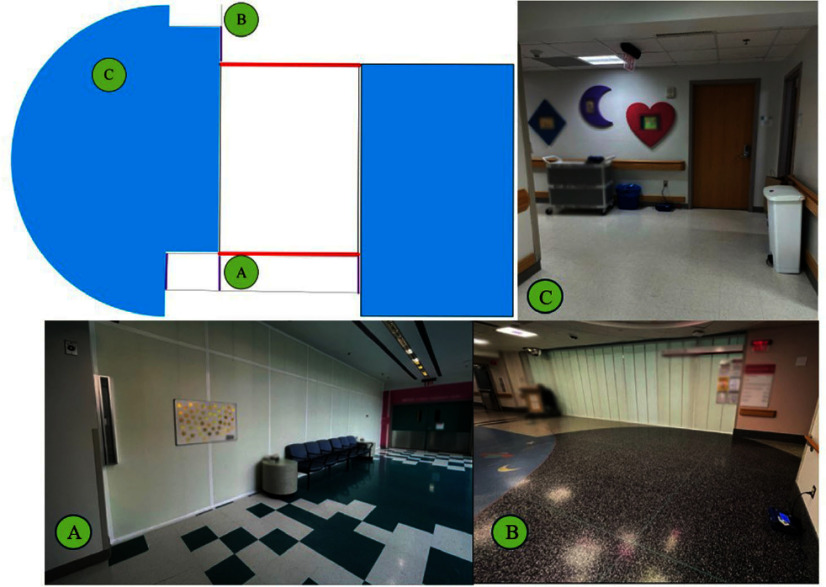

**Figure:**